# Measuring Actual eHealth Literacy Among Patients With Rheumatic Diseases: a Qualitative Analysis of Problems Encountered Using Health 1.0 and Health 2.0 Applications

**DOI:** 10.2196/jmir.2428

**Published:** 2013-02-11

**Authors:** Rosalie van der Vaart, Constance HC Drossaert, Miriam de Heus, Erik Taal, Mart AFJ van de Laar

**Affiliations:** ^1^University of TwenteDepartment of Psychology, Health & TechnologyEnschedeNetherlands; ^2^University Medical Center UtrechtDepartment of Corporate CommunicationsUtrechtNetherlands; ^3^Arthritis Centre TwenteEnschedeNetherlands

**Keywords:** eHealth, literacy, Internet, online, health information, Health 2.0, skills, health care

## Abstract

**Background:**

The Internet offers diverse opportunities for disease management, through information websites (Health 1.0) and interactive applications such as peer support forums, online consults, and insight into electronic medical records (Health 2.0). However, various skills are required to benefit from Health 1.0 and Health 2.0 applications for one’s own health, known as eHealth literacy.

**Objective:**

To study the eHealth literacy of patients with rheumatic diseases and the types of problems they encounter when using the Internet in relation to their disease.

**Methods:**

In two studies, patients were asked about their current disease-related Internet use and their eHealth literacy was observed during performance tests. In study 1, 15 patients (aged 39-74) performed 6 information-retrieval tasks on the Internet (Health 1.0). In study 2, 16 patients (aged 24-72) performed 3 Health 2.0 tasks on a hospital-based online Web portal and 2 Health 2.0 tasks on interactive websites. Participants were asked to think aloud while performing the assignments, and screen activities were recorded. Types and frequency of problems were identified by 2 independent researchers and coded into categories using inductive analysis.

**Results:**

Almost all patients in our studies had searched the Internet for information about rheumatic diseases in the past. Fewer patients had used Health 2.0 applications, but many were nevertheless enthusiastic about the possibilities from Health 2.0 applications after finishing the assignments. However, nearly all participants experienced difficulties, and a substantial number of participants were not able to complete all of the assignments. Encountered problems could be divided into 6 sequential categories: (1) operating the computer and Internet browser, (2) navigating and orientating on the Web, (3) utilizing search strategies, (4) evaluating relevance and reliability, (5) adding content to the Web, and (6) protecting and respecting privacy. Most severe difficulties occurred in levels 3 and 4—in formulating a search query, evaluating the source of the information, and in scanning a website for relevant information.

**Conclusions:**

Many patients have insufficient skills to properly use Health 1.0 and Health 2.0. Formulating proper search strategies and evaluating the found information caused problems among the majority of patients. Concerning Health 2.0, use and awareness of these applications is low and patients should be guided in the use of them. Our findings may contribute to the awareness of patients’ eHealth literacy problems among health professionals, and stress the importance of usability guidelines in Web design.

## Introduction

Since patients with chronic diseases are encouraged to become more empowered and to play a larger role in the management of their own disease, easily accessible health information is essential [1]. Currently, the Internet is one of the main sources of health information and research shows that many patients use it frequently [2,3]. Online access to health information is a positive development; studies have shown that people with chronic diseases who use the Internet have better self-care practices than those who do not [4,5]. With the improved Web technology (Web 2.0), the Internet has become more than an online encyclopedia. Not only can information be received from the Internet, but people can also add content to the Internet themselves. Health 2.0 is a term that is used for these interactive applications within health care [6]. This evolution of the Internet means that patients can communicate together online to share and find acknowledgement of their disease experiences through peer support forums [7], social network websites, and health care rating sites. Furthermore, hospitals are increasingly offering patients Interactive Health Communication Applications, which are Web-based portals that enable patients to participate online in their treatment, by communicating with care providers, monitoring symptoms using online diaries, and by accessing their electronic medical records. All these Health 2.0 applications have great potential and could change health care in a positive way [6,8,9]. Nevertheless, using the Internet in relation to health requires a certain level of eHealth literacy, which covers a diverse range of skills [10,11]. Norman defines eHealth literacy as “the ability to seek, find, understand, and appraise health information from electronic sources and apply the knowledge gained to addressing or solving a health problem” [11]. It should be noted, however, that this definition is limited to skills needed for Health 1.0 applications and that additional skills are needed for typical Health 2.0 applications [12].

A number of previous studies have shown that using the Internet to collect information requires skills on several levels. On a lower level, operational and navigation skills are essential—the competence to use a computer and an understanding of the World Wide Web and its multi-layer structure (including competencies to operate Internet browsers and search engines). On a higher level, people need skills to find and judge information, which requires the ability to generate relevant search queries, choose relevant information from the enormous amount of search results, and judge the reliability and validity of the information [13,14]. Research on Internet skills of people has so far focused on the general healthy population [13,15], and to a larger extent on adolescents and students [16-19]. Skills of patients with chronic diseases have not been studied yet, so it is unclear to what extent patients can benefit from the large amount of online information that is being offered. Additionally, studies up until now have not taken into account interactive Health 2.0 applications [12]. Using Health 2.0 applications asks for additional skills, such as being able to express oneself clearly in online social interactions, the ability to distinguish professional from non-professional advice [12], and protecting one’s privacy and respecting that of others when adding content to the Internet [6]. Due to the rapid developments on the Internet and the shift to Web 2.0 applications, these skills should be taken into account to measure the complete spectrum of eHealth literacy. The aim of this study was to gain an in-depth insight into Health 1.0 and 2.0 literacy skills of patients with rheumatic diseases.

## Methods

### Study Components

Two performance tests were conducted to investigate the skills of patients when using online information, communication, and participation sources with regard to rheumatic diseases. Study 1 was predominantly aimed at information retrieval through health-related websites and reading along on peer support forums (Health 1.0), study 2 was aimed at the use of interactive online applications (Health 2.0). In both studies, a qualitative design was used to get in-depth insight into patients’ strategies when using both kinds of applications. Patients were observed and were asked to think aloud [20] while performing various online assignments.

### Participants

Participants in study 1 were selected from an existing patient panel, which was initiated in cooperation between the University of Twente and Twente’s largest hospital, both located in Enschede, the Netherlands. Patients who are registered on this panel (n=146) are willing to volunteer in rheumatology research. A convenience sample from this panel was selected, based on attendance of these panel members at the research meeting introducing the upcoming study. Panel members that were present (n=30) were asked to fill out a form with their contact information if they were willing to participate in the study. Half of the panel members (15/30, 50%) filled out the form, and were subsequently called to explain the process of the study and to schedule an appointment. All appointments, except 2, took place at the university, to ensure that all participants were tested in the same environment. The 2 patients preferred to be interviewed at their home due to the travel distance. In study 2, participants were selected from the consult database of the rheumatology clinic of University Medical Centre, Utrecht. Participants who had a visit scheduled at the outpatient department of the clinic received an invitation letter by their nurse practitioner. Patients who were not able to speak Dutch and patients who needed an intensive infusion treatment during their hospital visit (which could cause limited mobility, tiredness, and nausea) were excluded. In total, 45 letters explaining the purpose and the process of the study were sent. The researcher called each recipient a few days after the letters were received to ask if they were willing to participate. Of the 45 patients, 17 (38%) gave their consent. An appointment for this study was scheduled on the same day as their existing upcoming appointment in the hospital. In both studies, patients were asked to sign an informed consent form at the beginning of the session, which included information on the recording, anonymity, and confidentiality of the data, and the possibility to end the session at any moment. In total, 15 patients participated in study 1 and 16 patients in study 2. One participant dropped out during study 2 because he felt uncomfortable in the test setting. After these sessions, data saturation was reached, meaning that no more new information of value could be obtained (no new problems occurred during the last 3 observations) [21].

### Procedure and Materials

The sessions started with a short survey which assessed demographic information (age, gender, and education), illness related information (diagnosis and disease duration), and Internet experience (amount of Internet use, years of Internet experience, self-perceived Internet skills, and usage of health-related applications on the Internet). The survey also contained a questionnaire on rheumatic-related physical problems when using a computer [22]. When all the items were completed, the practical component of the session started. In both studies, all participants used the same hardware, with the same settings. According to the thinking-aloud method [20], subjects were explicitly instructed to think aloud as they executed the assignments, which allowed the interviewer to get a better understanding of the cognitive processes the participant used to search and judge the information, and to formulate questions or messages. It was emphasized that the assignments were not to test the quality of participants’ Internet skills, but solely to observe how they used the Internet. The online assignments were recorded using Morae Recorder version 3.2.1 (TechSmith, Okemos, MI, USA), which captured images, sounds, and all screen activities. A description of all assignments is shown in Textboxes 1 and 2. In study 1, participants could search the Internet freely during the assignments and skills on several levels were needed to complete the assignments (see Textbox 1). Assignment 2 was the only exception in this study, where all patients were limited to performing the assignment on a single website (the website of the Dutch rheumatology association), which served as a reference to test patients’ operational skills of the computer and Web browser. In study 2, assignments 1, 2, and 3 had to be performed on a research account of a hospital-based Web portal, and assignments 4 and 5 on specific interactive websites (see Textbox 2). These assignments were specifically aimed at measuring Health 2.0 related skills, as patients were asked to add their own content. The assignments asked for skills in addition to information retrieval, such as expressing oneself in online social interactions, distinguishing professional from non-professional advice, and protecting one’s privacy and respecting that of others. The Health 1.0 assignments contained pilot tests investigating the relevance of the assignments to rheumatology patients, to ensure that the information-retrieval assignments reflected realistic scenarios. The Health 2.0 assignments were built based on results from previous studies [2,23], which highlights information that patients would find relevant and useful. We therefore only asked nurse practitioners to help us frame the scenarios for the Health 2.0 assignments. In both studies, the order of the assignments was randomized for each participant, because a learning effect was expected during the assignments. By randomizing the sequence of the assignments, this effect would not occur at the same assignments for every patient. At the end of study 2, participants were asked if they would use the interactive application in the future, what they would use it for, and how they would take privacy issues into account, after each assignment. These interviews were video-recorded with the Morae Recorder as well.

**Description of Health 1.0 Assignments in Study 1.**

1. Formulate a disease-related question you have searched for in the past, and show how you would approach this on the Internet.
2. Open a well-known Dutch rheumatology website [24] and perform the following assignments: find a specific page using the menu structures, download a PDF file, close the additional window, go one page back, use the search engine to search for “osteoarthritis”, open the fourth search result and save that page using the favorites bar.
3. You have had sore wrists and hands for a while and you think it might be osteoarthritis. Retrieve the symptoms of osteoarthritis on the Internet and mention 4 of them.
4. You are using MTX medication for your rheumatic symptoms, but as a side effect you feel nauseated. Retrieve 3 tips from fellow patients on a patient support forum on how to lessen nausea as a side effect from this medication.
5. You are experiencing sore feet due to your rheumatic symptoms and you want to buy adapted shoes to relieve the pain. Find 4 key issues to consider when buying adapted shoes.
6. You would like some advice on how to exercise properly in spite of your arthritis. Find a physical therapist in your neighborhood that is familiar with therapy for rheumatic diseases.

**Description of Health 2.0 Assignments in Study 2.**

1. Use your electronic medical record to: (a) find and interpret your latest lab results and compare them to your previous values, and (b) to interpret the accompanying treatment plan.
2. Monitor disease symptoms by: (a) filling out a disease diary for one day, and (b) interpret two previous diaries.
3. Use an e-consult (electronic or online consult) application to: (a) find and interpret a closed e-consult, and (b) to write a new e-consult in which you ask advice on how to bring your medication with you on vacation to Morocco.
4. Open a peer support forum [25] and: (a) retrieve 2 tips from fellow patients on nausea as a side effect from MTX medication, and (b) add your own tip on this subject.
5. Open a health care rating website [26], find your hospital and: (a) read 2 reviews, and (b) add your own review about the hospital (you do not have to send it).

### Data Analysis

Descriptive analyses of patients’ socio-demographics, health characteristics, health-related Internet use, and rheumatic-related physical problems when using a computer were performed with SPSS Statistics, version 20.0 (IBM SPSS Inc, Chicago, IL, USA). Performances on the assignments were analyzed using Morae Manager version 3.2.1 (TechSmith, Okemos, MI, USA). In study 1, 2 researchers inductively developed a coding scheme in which all patients’ actions were independently coded and categorized into main categories and further into subcategories [27]. To get an indication of the number of participants that experienced problems in each category, we counted the number of patients that experienced each defined problem in a specific assignment, and we counted the number of individual participants that experienced each defined problem in the total test (see Table 4, last column). The difficulty of the assignments was then accessed based on the number of participants that experienced more than one problem in each assignment (see Table 4, bottom row). Study 2 used the same coding scheme as study 1 but was expanded to account for Health 2.0 literacy problems. In both studies, 3 other outcomes per participant were measured. First, completion of the assignment was registered as “completed independently”, “completed with help” (when a hint or intervention from the research leader was needed), or “not completed”. The research leader only provided assistance if a patient said he or she was about to give up on the assignment. If the patient did not say this, but was clearly lost or frustrated, the research leader asked the patient if he/she would have quit during a similar search at this point if he/she were at home. If the answer was yes, the research leader provided some assistance. Due to the variation in determination among patients to finish the assignments independently, the moment until assistance was offered varied between 1 minute and 20 minutes. Second, the time needed to perform the assignment was registered, but only for the participants that completed the assignment. Finally, the performance was registered, which was scored as “good”, “reasonable”, or “poor”, according to the skills participants used to execute the assignment. The performance was rated as “good” when both researchers agreed that the operational skills and strategic skills were adequate, “reasonable” if not all skills were shown convincingly, and “poor” if patients showed severe problems on all skill levels. The interviews in study 2 were transcribed verbatim and coded inductively. Differences in codes and the distribution among the codes were discussed between the researchers before and during the study for each patient until consensus was reached. In case of doubts (which occurred in a few occasions), a third independent researcher was involved to come to a conclusion.

## Results

### Participants

Participants’ characteristics are shown in Table 1. The majority of the participants were female, and the mean age was 56.4 and 48.6 for study 1 and 2, respectively. Most participants were diagnosed with rheumatoid arthritis and had the disease for several years. Table 2 shows participants’ current, disease-related Internet use. Most participants used the Internet on a regular basis and rated their own Internet skills as “good”. The large majority of participants had searched for online disease-related information (28/31, 90%). Some Health 2.0 applications were used by a substantial group of participants, such as using health care reviews (10/31, 32%), ordering medications online (10/31, 32%), or sending an e-consult (9/31, 29%). However, fewer participants used other Health 2.0 applications, such as adding content to a peer support forum (4/31, 13%) or posting a health care review (1/31, 3%).

### Physical Problems When Operating the Computer

Computer-related problems caused by physical impairments in the questionnaire were reported by 7 participants in study 1 (7/15, 47%), and 6 participants in study 2 (6/16, 38%, data not shown). Problems were related to their chair (8/31, 26%, mainly finding a comfortable chair, or a good position in the chair), keyboard (8/31, 26%, mainly pressing individual keys, finding a good position for their hands, and becoming stiff or tired from typing), the mouse (7/31, 23%, mainly double clicking, finding a good position for their hand and becoming stiff or tired from using the mouse), and the monitor (7/31, 23%, mainly finding a good position and getting tired from looking at the screen). During the performance of the assignments, 3 participants mentioned difficulties due to physical impairments (3/31, 10%); 1 participant had to stand up for a while to stretch her legs and 2 participants mentioned they had trouble typing, 1 due to a wrist splint.

**Table 1:** Participants’ socio-demographics and illness related information in the 2 studies.

Characteristic		Study 1 (n=15) n (%)	Study 2 (n=16) n (%)
**Gender**			
	Male	3 (20)	3 (19)
	Female	12 (80)	13 (81)
**Age**			
	Mean (SD)	56.4 (10.5)	48.6 (14.2)
	Range	39-74	24-72
**Education**			
	Low	4 (26)	4 (25)
	Middle	2 (13)	6 (38)
	High	9 (60)	6 (36)
**Diagnosis**			
	Rheumatoid Arthritis	10 (67)	12 (75)
	Osteoarthritis	3 (20)	0 (0)
	Ankylosing Spondylitis	0 (0)	3 (19)
	Other rheumatic disease	3 (20)	1 (6)
	Unknown	1 (7)	0 (0)
**Years since diagnosis**			
	Mean (SD)	13.5 (13.1)	9.1 (7.4)
	Range	3-52	2-25

**Table 2:** General and disease-related Internet use in the 2 studies.

			Study 1 (n=15) n (%)	Study 2 (n=16) n (%)
**Amount of Internet usage**				
	(Almost) everyday		11 (73)	14 (88)
	Several days a week		3 (20)	1 (6)
	About 1 day a week		1 (7)	0 (0)
	(Almost) never		0 (0)	1 (6)
**Internet experience in years**				
	≥ 5 years		13 (87)	14 (88)
	< 1 year		2 (13)	2 (12)
**Self-assessed Internet skills**				
	Excellent		0 (0)	1 (6)
	Good		6 (40)	6 (38)
	Average		3 (20)	3 (19)
	Reasonable		6 (40)	4 (25)
	Poor		0 (0)	2 (13)
**Number of respondents who have ever online:**				
	searched for information on rheumatic diseases		13 (87)	15 (94)
	read a peer support forum or social media website		8 (53)	8 (50)
	read a health care review		5 (33)	5 (31)
	ordered medications at the pharmacy		4 (27)	6 (38)
	asked a question to their health care provider		2 (13)	7 (44)
	monitored disease symptoms		5 (33)	0 (0)
	logged onto their own electronic medical record		2 (13)	3 (19)
	scheduled an appointment with their health care provider		4 (27)	0 (0)
	posted a message on a peer support forum or social media website		1 (7)	3 (19)
	shared personal medical information with others		2 (13)	1 (6)
	joined an online self-management course		1 (7)	0 (0)
	posted a health care review		1 (7)	0 (0)

### Study 1

#### Execution of Health 1.0 Assignments and Problems Encountered

Table 3 shows that the first 3 information-retrieval assignments (retrieving information someone had previously searched for, performing operational assignments, and finding 4 symptoms of osteoarthritis) went rather well for most participants. The last 3 assignments (searching for tips from fellow patients, finding key aspects of adapted shoes, and finding a specialized physiotherapist in the neighborhood), however, were more difficult. These assignments could not be completed by almost half of the participants, many performed poorly in searching for the right answer and the median times to complete these assignments were greater than the first 3 tests.

Observed problems among participants when performing the 6 Health 1.0 assignments could be coded into 4 categories (see Table 4): (1) operating the computer and Internet browser, (2) navigating and orientating, (3) utilizing search strategies, and (4) evaluating relevance and reliability of Web content.

**Table 3:** Completion, performance, and time needed for completion on the Health 1.0 assignments (n=15).

Assignment		1^a,g^ n (%)	2^b,h^ n (%)	3^c^ n (%)	4^d^ n (%)	5^e^ n (%)	6^f^ n (%)
**Completion**							
	Independently	11 (79)	8 (57)	12 (80)	7 (47)	8 (53)	8 (53)
	With help	0 (0)	1 (7)	0 (0)	2 (13)	0 (0)	0 (0)
	Not completed	3 (21)	5 (36)	3 (20)	6 (40)	7 (47)	7 (47)
**Performance**							
	Good	4 (29)	9 (64)	7 (47)	4 (27)	6 (40)	5 (33)
	Reasonable	7 (50)	3 (21)	6 (40)	4 (27)	4 (27)	5 (33)
	Poor	3 (21)	3 (21)	2 (13)	7 (47)	5 (33)	5 (33)
**Duration to complete the assignment (seconds)** ^**i**^							
	Median	177	192	225	563	311	268
	Minimum	60	103	115	274	247	186
	Maximum	848	234	488	1095	512	524

^a^retrieve previous searched disease information

^b^perform operational assignments

^c^search for 4 symptoms of osteoarthritis

^d^search for 3 tips from fellow patients on MTX side effects

^e^retrieve 4 key aspects when buying adjusted shoes

^f^find a physiotherapist specialized in rheumatic diseases in your neighborhood

^g^A participant had never searched for information on her rheumatic disease (n=14).

^h^A mistake occurred due to a change in the texts on the particular website that was used. This change in text occurred between the time of the pilot study and the first official session and was therefore, unfortunately, discovered during the first session. Therefore, the data of participant 1 could not be used (n=14).

^i^The times of participants who did not complete the assignment were not included.

**Table 4:** Problems and number of participants experiencing those problems in Health 1.0 assignments (n=15).

Assignment		1^a,g^ n (%)	2^b,h,i^ n (%)	3^c^ n (%)	4^d^ n (%)	5^e^ n (%)	6^f^ n (%)	Total^j^ n (%)
**Operating the computer and Internet browser**								
	Operating the keyboard/locating keys	2 (14)	1 (7)	0 (0)	1 (7)	1 (7)	0 (0)	5 (33)
	Controlling the mouse/clicking the mouse	4 (29)	3 (21)	2 (13)	2 (13)	3 (20)	1 (7)	6 (40)
	Using of the URL bar to open a Web address	1 (7)	8 (57)	0 (0)	0 (0)	0 (0)	0 (0)	9 (60)
	Losing track of the cursor	0 (0)	1 (7)	1 (7)	2 (13)	2 (13)	1 (7)	6 (40)
	Closing the Internet browser	3 (21)	1 (7)	3 (20)	2 (13)	3 (20)	3 (20)	5 (33)
	Using and closing more windows	0 (0)	4 (29)	0 (0)	2 (13)	0 (0)	1 (7)	6 (40)
	Using the scroll bar	1 (7)	0 (0)	0 (0)	0 (0)	1 (7)	0 (0)	2 (13)
	Participants with > 1 problem per assignment	2 (14)	5 (36)	2 (13)	2 (13)	2 (13)	1 (7)	6 (40)
**Navigating and orientating**								
	Using and understanding a PDF file	2 (14)	0 (0)	0 (0)	1 (7)	0 (0)	0 (0)	2 (13)
	Keeping orientation on a website	0 (0)	1 (7)	2 (13)	2 (13)	2 (13)	1 (7)	4 (27)
	Using dropdown lists	1 (7)	9 (64)	0 (0)	0 (0)	1 (7)	1 (7)	9 (60)
	Recognizing and using Web links	2 (14)	3 (21)	2 (13)	2 (13)	3 (20)	2 (13)	7 (47)
	Using a search engine within a website	0 (0)	3 (21)	0 (0)	0 (0)	0 (0)	0 (0)	3 (20)
	Participants with > 1 problem per assignment	0 (0)	3 (21)	2 (13)	1 (7)	3 (20)	1 (7)	5 (33)
**Utilizing search strategies**								
	(Too) broad search query	6 (43)	n/a	5 (33)	12 (80)	6 (40)	9 (60)	15 (100)
	(Typing) errors in search query	4 (29)	5 (36)	7 (47)	3 (20)	3 (20)	2 (13)	14 (93)
	Choosing a relevant search result	5 (36)	n/a	8 (53)	9 (60)	7 (47)	7 (47)	13 (87)
	Keeping focus on the needed information	5 (36)	0 (0)	2 (13)	6 (40)	5 (33)	7 (47)	11 (73)
	Participants with > 1 problem per assignment	8 (57)	0 (0)	8 (53)	12 (80)	5 (33)	9 (60)	14 (93)
**Evaluating relevance and reliability**								
	Not checking the source of the information	8 (57)	n/a	14 (93)	14 (93)	13 (87)	12 (80)	14 (93)
	Opening only one search result	4 (29)	n/a	11 (73)	13 (87)	7 (47)	7 (47)	15 (100)
	Searching in commercial websites	2 (14)	n/a	2 (13)	2 (13)	6 (40)	4 (27)	11 (73)
	Scanning a website for relevant information	3 (21)	0 (0)	5 (33)	7 (47)	4 (27)	3 (20)	12 (80)
	Selecting a relevant answer	3 (21)	n/a	3 (20)	4 (27)	8 (53)	6 (40)	11 (73)
	Participants with > 1 problem per assignment	6 (43)	n/a	14 (93)	14 (93)	12 (80)	12 (80)	14 (93)

^a^retrieve previous searched disease information

^b^perform operational assignments

^c^search for 4 symptoms of osteoarthritis

^d^search for 3 tips from fellow patients on MTX side effects

^e^retrieve 4 key issues when buying adjusted shoes

^f^find a physiotherapist specialized in rheumatic diseases in your neighborhood

^g^A participant had never searched for information on her rheumatic disease (n=14).

^h^A mistake occurred due to a change in the texts on the particular website that was used. This change in text occurred between the time of the pilot study and the first official session and was therefore, unfortunately, discovered during the first session. Therefore, the data of participant 1 could not be used (n=14).

^i^This assignment was aimed at operational and navigation skills, therefore most strategic skills were not applicable (n/a)

^j^The number of participants that experienced this particular problem at least once during the complete tests (6 assignments). The numbers in the rows do not add up, since one patient could experience the same problem during several assignments.

#### Operating the Computer and Internet Browser

The first category of problems concerned operating the computer and Internet browser. Participants experienced difficulties when using the hardware of the computer, and when using the main buttons and fields in the Internet browser. Concerning the hardware of the computer, 5 participants experienced problems when using the keyboard, mainly to locate keys. Difficulties operating the mouse were experienced by 6 participants during one or more assignment, especially keeping control over movements of the mouse, and double clicking on buttons. Regarding the use of the Internet browser, 9 participants did not use the address bar when asked to navigate to a particular Web address, but they used the search engine to type in the Web address. One participant asked, “If I type something in Google, do I have to type ‘.nl’ at the end?” [Female, 62 years old]. Six participants lost track of the cursor when they wanted to type something in a field, which would cause confusion (eg, “Where am I?” [Female, 39 years old]). The buttons in the browser that caused the most problems were the close button, the multiple tabs, and the scroll bar. Five participants did not know how to close the Internet browser during one or more assignments after an assignment had ended. Two of them continuously clicked on the ‘back’ button to go back to the beginning of the search.

Is it necessary to do what I’m doing now? Should I click this button (“back”) until the arrow disappears? Or can I just close it all at once, without erasing anything?

Female 62 years old

Another participant minimized the window instead of closing it, and one participant tried clicking on the stop button of the address bar. In assignment 2, where patients were explicitly asked to open and close a second tab, 4 participants were not able to fulfill the task. Participants did not seem to understand that they had 2 separate tabs open, parallel to each other, so they were not aware that they could close 1 tab, while keeping the other open. Problems associated with the scroll bar included lose of control over the scroll function, which caused the text to speed by. Overall, operational problems were not assignment-specific and did not occur too often for most patients; 6 participants experienced problems repeatedly.

#### Navigating and Orientating

The second category of problems concerned navigating and keeping orientation in the Internet browser and on websites. Overall, the multilayer structure of the Internet caused problems, which was often observed when a PDF file was opened. A few participants did not understand that a PDF file is not a website, and that a PDF file has a different navigation structure, in which scrolling is much more important and Web links often do not exist. Furthermore, because websites often combine navigation structures (such as navigation trails [ie, breadcrumbs], navigation buttons/tabs, or internal hyperlinks), keeping orientation sometimes caused difficulties among patients. The different navigation structures should enable visitors to retrieve webpages via different routes. However for 4 participants this caused disorientation in one or more assignments. These patients did not notice that the different navigation structures led to the same website and they lost track of their location in the Web page, or they thought the page was still loading, while they were actually already on it. When navigating through a website, dropdown lists, Web links and search engines were often not used as intended. Not all participants understood that dropdown lists function as a “hidden” menu, therefore, the mechanism of the list was problematic for many of them. Particularly in a double dropdown list, where a dropdown list unfolds into another dropdown list (which was used in assignment 2), 9 participants experienced difficulties, since they were not able to click on a button before the list closed again. Seven participants experienced several problems with Web links in one or more assignments, for example not recognizing a relevant link or clicking on a word that was not a link (eg, “Shouldn’t there be a little hand here?” [Female, 52 years old]). Interestingly, a small group of participants generated a large amount of the problems encountered during navigation and orientation. These were the same participants that experienced the most operational problems.

#### Utilizing Search Strategies

A third category of problems was observed in participants’ search strategies. The majority of these problems occurred in the first stage of the search where the search query was formulated. Often participants started searching with only one query, which was too broad to complete the assignment. A few participants did not seem to understand that they could adjust or expand their query and they blamed the computer for not being able to find the right information. A second major problem in formulating a search query was typing and spelling errors.

When I click on this (search result) I expect to find the right information. That is what I expect from the computer.

Female, 63 years old

At home I would get my dictionary.

Female, 39 years old

Not all participants were aware of their mistakes and did not use the autocorrect function from Google, which led to flawed search results, or very few search results. A frequent problem in the second stage of participants’ search strategies occurred in selecting a website from the list of search results. Many participants randomly chose one of the first search results on top of the page. When selecting a search result, they did not seem to look at the URL or the description of the site just below to get an indication of the content of the website, “I just try the first one.” [Female, 45 years old]. Only 1 participant in a single assignment looked further than the first page with search results. One participant mentioned that, “the most important results are shown on the first page anyway” [Male, 62 years old], however, some participants did not seem to realize that the search results extended after the first page. The last problem in applying a logical search strategy was the loss of focus on the required information. Patients became distracted by other information they found interesting (eg, “Here I read osteoarthritis is hereditary, my sister recently has sore shoulders as well” [Female, 74 years old]). Overall, all participants experienced difficulties in their search strategies at some point. However, most participants showed a learning curve and altered their search strategies as the study progressed, while 4 patients did not seem to be aware of their mistakes and used the same trial-and-error method in several assignments.

#### Evaluating Relevance and Reliability

The last category of observed problems, evaluating relevance and reliability of Web content, caused the largest number of problems. Almost none of the participants consciously checked the source or the topicality of the information. No one verified the information they found on one website with information from another website. Participants only opened a second search result when they could not find the correct answer in the first one. However, not all participants seemed to understand that they could go back to the list of search results to explore a different website. One woman was searching on a peer support forum for people suffering from hyperhidrosis (excessive sweating), instead of a peer support forum for rheumatoid arthritis, but did not go back to the search results to something relevant. Eventually she asked the research leader, “Are you sure the information can be found on a patient forum?” [Female, 39 years old]. Many participants did not seem to be aware of different sources of information. Only 3 participants made a remark about the occurrence of sponsored hits at the top and on the right side of the search results. Furthermore, when searching on a website, many participants did not scan the website for relevant information to fulfill the assignment. Participants would select buttons with irrelevant titles and read webpages verbatim without considering the relevance of the information. In the peer support forum, this occurred regularly. One participant selected a random topic on rheumatoid arthritis “keep having knee pains” and read all the messages out loud, even though they were not relevant for the assignment. In fact, she commented that, “there are so many messages here, and you need to work through them all. What a waste of time” [female, 59 years old]. Many participants did not give different value to the information provided by different sources, such as a commercial company, a peer support forum, or a national foundation.

### Study 2

#### Execution of Health 2.0 Assignments and Problems Encountered

In study 2, the majority of the participants completed all assignments, most of them without help (Table 5). Not all participants were able to start and complete all assignments, because they were tired after completing 3 or 4 assignments, or because they had to leave for their doctors’ consult. Ten participants (10/16, 63%) started all assignments and 9 participants (9/16, 56%) completed all assignments. Assignments 2 (filling out a diary), 3a (writing an e-consult) and 5b (posting a health care review) were the most difficult for participants as these assignments required addition of content to the Web. The minimum and maximum duration varied widely between participants within each assignment, which was an indication of the different skill-levels between participants. From the interviews, we found that almost all participants had no experience with the assignments. No one had monitored their disease symptoms before or posted a health care review. One patient (1/16, 6%) had previously sent an e-consult, 3 patients (3/16, 19%) had posted a message on a peer support forum, and 3 patients (3/16, 19%) had consulted their electronic medical records before. Nevertheless, after finishing the assignments, the patients perceived the e-consult and access to the electronic medical record components especially valuable. Eleven patients (11/16, 67%) would like to use an e-consult in the future, and 14 patients (14/16, 89%) reported they would open their electronic medical records at home.

**Table 5:** Completion, performance, and time needed on the Health 2.0 assignments (n=16).

Assignment		1a^a^ n (%)	1b^b^ n (%)	2a^c^ n (%)	2b^d^ n (%)	3a^e^ n (%)	3b^f^ n (%)	4a^g^ n (%)	4b^h^ n (%)	5a^i^ n (%)	5b^j^ n (%)
Number of participants who started the assignment, n		15	15	16	16	15	15	12	10	15	15
**Completion**											
	Independently	12 (80)	14 (93)	10 (63)	11 (69)	9 (60)	13 (87)	10 (83)	8 (80)	12 (80)	10 (67)
	With help	3 (20)	1 (7)	3 (19%)	2 (13%)	2 (13)	1 (7)	0 (0)	1 (10)	1 (7)	0 (0)
	Not completed	0 (0)	0 (0)	3 (19%)	3 (19%)	4 (27)	1 (7)	2 (17)	1 (10)	2 (13)	5 (33)
**Performance**											
	Good	11 (73)	11 (73)	8 (50)	11 (69)	9 (60)	13 (87)	6 (50)	7 (70)	10 (67)	8 (53)
	Reasonable	1 (7)	3 (20)	4 (25)	0 (0)	1 (7)	0 (0)	4 (33)	1 (10)	5 (33)	4 (27)
	Poor	3 (20)	1 (7)	4 (25)	5 (31)	5 (33)	2 (13)	2 (17)	2 (20)	0 (0)	3 (20)
**Duration** ^**k**^											
	Median	151	119	265	66	163	115	270	262	159	226
	Minimum	57	24	92	27	71	24	128	156	101	108
	Maximum	746	263	782	156	544	723	629	1406	674	672

^a^use electronic medical records to find and interpret lab results

^b^use electronic medical records to interpret treatment plan

^c^monitor disease symptoms by filling out a diary

^d^monitor disease symptoms interpreting 2 previous diaries

^e^use e-consult to write a new e-consult

^f^use e-consult to read a previous e-consult

^g^use a peer support forum to find 2 tips

^h^use a peer support forum to add your own tip

^i^use a health care rating website to read reviews

^j^use a health care rating website to post a review.

^k^time is in seconds and the time of participants who did not fulfill the assignment is not included in this median

Many of the problems encountered in study 2 corresponded to those found in study 1. However, it should be noted that in study 2, the participants were somewhat assisted, as they were guided to specific websites. Therefore we restricted the report of results in study 2 to an overview of the observed additional problems in Health 2.0 assignments in category 4 (evaluating relevance and reliability), category 5 (adding personal content to the Web in assignments 2a, 3a, 4b, and 5b, see Table 6), and category 6 (protecting and respecting privacy).

#### Evaluating Relevance and Reliability

A new subcategory in evaluating relevance and reliability, which was added to the findings of study 1 was reading and interpreting the information correctly (not shown in Table). This category had to be added since information on the specified Web portal was always reliable, and mostly relevant, but participants still had to read and interpret the information correctly. This was of particular concern in assignment 1, in which participants had to interpret several lab results and compare them to previous values. Reading and interpreting the information correctly caused problems among 7 participants, mostly because they had difficulties to see which lab results were the most recent, and because they did not take into account the given information about reference values. Four participants assumed that increased lab values were always bad (eg, “The levels are higher than the last time, that is bad, right?” [Female, 24 years old]), and 3 participants reported that they did not know if the values worsened or not (eg, “I’m no expert in this; I have never studied these things.” [Female, 65 years old]). Only 1 participant reported she would be worried if those were her personal data and she would call her doctor immediately. The other participants reported that they probably would have heard it from their rheumatologist if anything was wrong, or they would ask about it in their next consult, call their care provider, or send an e-consult.

**Table 6:** Health 2.0 problems with adding personal content to the Web, including amount of participants experiencing those problems (n=16).

Assignment		2a^a^ n=16 n (%)	3a^b^ n=15 n (%)	4b^c^ n=10 n (%)	5b^d^ n=15 n (%)	Total^e^ n=16 n (%)
**Number of participants who experienced problems associated with adding personal content to the Web**						
	Using proper fields for adding data	3 (19)	6 (40)	5 (50)	3 (20)	10 (63)
	Using capital letters and punctuation marks	11 (69)	8 (53)	4 (40)	2 (13)	13 (82)
	Spelling	5 (31)	2 (13)	4 (40)	2 (13)	8 (50)
	Using appropriate header and sender information	n/a^f^	7 (47)	6 (60)	0 (0)	10 (63)
	Formulating a question or message	4 (25)	7 (47)	2 (20)	4 (27)	10 (63)
	Participants with > 1 problem per assignment	6 (38)	10 (67)	3 (30)	5 (33)	14 (88)

^a^monitor disease symptoms filling out a diary

^b^use e-consult to write a new e-consult

^c^use a peer support forum to add your own tip

^d^use a health care rating website to post a review

^e^The number of participants which experienced this particular problem during the complete test (5 assignments). The numbers in the rows do not add up because one patient could experience the same problem during several assignments.

^f^using headers and sender information was not applicable in assignment 2

#### Adding Personal Content to the Web

Difficulties with adding personal content to the Web existed in several subcategories and were related to the correct formulation of the message or question to be placed on the Web (Table 6). First, there were several practical issues in adding content to the Web. Some participants experienced difficulties in using the proper fields for their information. For example, when writing an e-consult, 1 participant wrote her question in the subject field. Other participants forgot to fill in a subject for the e-consult, or an addressee to send the e-consult to. As a result, the send button did not become active. Not all participants understood this, and 3 participants thought that the e-consult was sent anyway (eg, “He is sending my message now, right?” [Female, 24 years old]). Subsequently, there were many minor problems with the actual writing of a message, namely spelling errors, lack of punctuation, and capital letters. These errors could influence the readability of the content and how well the message was understood. Third, several participants found it difficult to reflect on whom the reader of their message would be, and what tone would be appropriate. For example, when writing an e-consult to their care provider in the personal Web portal, it would be convenient to use a header and conclude with a name, surname, and maybe a patient number. However, when writing on a peer support forum, messages can be more informal and one might explicitly not conclude the message with a name (or use a nickname) for privacy matters (see next section). Some participants did not seem to be aware of this difference. Lastly, and perhaps most importantly, half of the participants showed problems in the actual formulation of a message or a question. They were not able to reflect on what information was necessary for the reader to understand their message or question. Also, participants used incomplete sentences in their messages (eg, “Sometimes have feeling in rheumatology that things are intertwined, mb too busy” [Female, 35 years old]), or asked an irrelevant question (eg, “Don’t I need medication because it’s so warm over there?” [Male, 48 years old]).

#### Protecting and Respecting Privacy

The last category of observed problems comprised of the protection of one’s own privacy and respecting that of others. During the assignments, it was difficult to code how participants handled their privacy, because the assignments were fictional and participants did not actually have to save or send their added content. Very few participants mentioned their privacy during the assignments, therefore, the findings presented here are based on what participants mentioned during the assignments and on the interviews afterwards. Concerning access to electronic medical records or using e-consult, no one made a remark about privacy during the assignment or in the interview afterwards. Apparently, all participants were confident that their data was secure in the Web portal. Nevertheless, 3 participants would not monitor their disease symptoms due to privacy issues, although this assignment was performed on the same Web portal. Two participants did not like the idea of putting all their information online for anyone to view and access.

I am an Internet user from a previous generation; I don’t put down my whole life story online. It might go wrong and then all my information is out in the open.

Female, 35 years old

One participant read the accompanying text when filling out her diary, and saw that her care providers had access to the monitored data as well, which discouraged use.

Oh, my care provider is reading along? For me that’s a reason not to use it!

Female, 57 years old

With regard to the 2 assignments outside of the Web portal (using a peer support forum and a health care rating site), more participants seemed aware of privacy issues. A message was added to the peer support forum by 1/9 (11%) participants, 4 mentioned only their surname, and 5 participants did not sign their message at all. However, in 8 occasions, it was not clear if this was on purpose or not, because participants did not explain their choice. Only 1 participant specifically said that they did not want to be judged by the readers, and therefore left out their name. Of these 9 participants, 4 would use a peer support forum in the future. All these participants reported that they would only write general information about themselves and that they would never write anything about others without their consent. Out of the 10 participants that filled in a health care review, 8 would use the website again in the future. However, only 2 participants would use the website to complain about a care provider, when a mistake was made, “I would only report it if a mistake was made, for I would hope to prevent that for someone else” [Female, 45 years old]. The other participants would not use the website to place a negative review because they would rather speak to their care provider in person about the issue and do not want to negatively sway the opinion of others.

### Relationship Between Patient Characteristics and eHealth Literacy

We explored if there were any correlations between the performance on the assignments, patients’ age, level of education, and perceived Internet skills (Multimedia Appendix 1). We found that patients who are higher educated, younger, and have higher self-perceived Internet skills, on average completed more assignments independently, performed better, and encountered fewer problems. However, these data should be interpreted with care, as the sample size was small.

## Discussion

In these 2 studies, a representative sample of patients with rheumatic diseases performed Health 1.0 and Health 2.0 assignments on the Internet. While a substantial number of patients experienced physical uneasiness when using the computer (eg, stiffness and tiredness) for the questionnaire, only 3 participants mentioned actual physical problems during the assignments. Nevertheless, using the Internet for health-related searches for a restricted amount of time seemed to be feasible for most participants. Furthermore, our results showed that a substantial group of patients were not able to fully use disease-related Internet applications for their own benefit. Problems in Health 1.0 information retrieval were found in 4 categories: (1) operating the computer and Internet browser, (2) navigating and orientating, (3) utilizing search strategies, and (4) evaluating relevance and reliability, which corresponds largely with categories found in a previous study by Van Deursen and Van Dijk among healthy people [28]. About one-third of the participants in our study had severe problems in operating the computer, the Internet browser, and in navigating and orientating on the Web. While these problems were often overcome, they did cause a substantial amount of inefficiency and frustration, withholding participants from fully using all the options the computer and Internet offers. Moreover, the more complex information and evaluation skills caused frequent problems for most of the patients. Many struggled with choosing a relevant search query, selecting a reliable search result, and browsing a website to find the right answer to a specific question. It seemed that a substantial part of the sample was using a trial-and-error method for searching the Internet. Strikingly, only 3-4 participants out of 15 were critical about the websites they visited and the information they retrieved from the Internet. The remaining participants did not seem to be aware of the source of the information, who exploited the website they searched on, and when the information was last updated. This is worrisome, since previous research studies have shown that many rheumatology-related websites provide unreliable information [29].

Although studies have been conducted to evaluate particular Health 2.0 applications [30,31], to our knowledge, no previous studies have been performed on Health 2.0 literacy of patients with chronic diseases and their ability to perform a variety of Health 2.0 assignments. This approach has enabled us to study Health 2.0 skills rather than evaluating the usability of a single application. During the assignments, we observed problems with operation, navigation, and information skills that corresponded with problems found in the Health 1.0 applications. However, since we provided patients with the direct website of the Health 2.0 applications, the Health 1.0 skills were not fully examined in this part of the study and the focus was on specific Health 2.0 problems. Most patients had little or no experience in using services to communicate with other patients, care providers, or with checking their own health status online, which corresponds with previous research among rheumatology patients [2]. Problems in doing so were mostly found in evaluating relevance and reliability (category 4) and in 2 additional categories: (5) adding personal content to the Web, and (6) protecting and respecting privacy. When adding personal content, several patients had trouble with using the content fields correctly, formulating a message and writing it down properly, and keeping in mind who the readers of the message will be. With reference to privacy issues, participants often mentioned being reluctant to add content to the Web. It was difficult for the participants to reflect on the reader of their information and what impact it would have on privacy when posting a message. Overall, it seemed that due to a lack of experience in online communication, many patients were insecure about when and how to use Health 2.0 applications. This lack of Health 2.0 use was seen in previous research as well [32]. Interpreting personal health records caused some problems, mostly because patients were not able to locate the relevant information and to put the information in the right context. This was partly due to incorrect interpretation of numbers, which also relates to numeracy skills [33]. Problems with interpreting electronic medical records are concerns that health professionals have previously reported [34,35]. Nevertheless, the action that patients would take in reaction to their personal data was generally appropriate. Keeping patients’ records clear and limited to the essence of the content would presumably overcome most of the observed difficulties [35]. After finishing the Health 2.0 assignments, many patients were enthusiastic about the possibilities the Internet could provide, and to become more involved in their own health care process, especially by using the applications that were provided by their own hospital. Two thirds of all participants would like to use e-consult in the future, and almost all patients reported they would open their electronic medical records at home, now that they have seen the service. Many patients were simply unaware of their options and/or anxious to use them themselves before the study. Therefore, patients need to be guided and encouraged to use Health 2.0 applications, and they should be informed by care providers about the privacy disclosures in such applications.

A limitation of our studies was the research setting in which participants performed the assignments. Although we aimed to formulate assignments that were relevant to patients with rheumatic diseases and stressed that the study was not an exam, patients were probably more nervous than if they were in a natural setting. Participants were also probably focused on completing the assignments quickly, which could influence the quality with which they performed. Furthermore, in several Health 2.0 assignments, patients were asked to spontaneously formulate a fictive question or message, which turned out to be difficult for some participants and might have complicated the assignment. Nevertheless, our studies demonstrate that most patients have considerable problems with using the Internet for health-related purposes. Although our studies were restricted to patients with rheumatic diseases, we feel that our results are generalizable for other health conditions, especially as only a minority of the participants’ perceived physical problems during the tests. Moreover, the assignments used in our performance tests (eg, using e-consults, health care rating sites and peer support forums) might be relevant to patients with different conditions. Because of the qualitative nature of our studies, we cannot draw strong conclusions on the most frequent eHealth literacy problems that patients encounter, and on which groups of patients encounter most problems. However, our studies showed that the majority of participants experienced difficulties on several levels, even though our research population was heterogeneous in age, education level, and had quite some experience in using the Internet. Previous research has shown that a higher education does not guarantee better Internet skills [36,37] and other studies among higher educated populations confirm these results. For example, Hughes et al [38] showed that doctors often choose their search results based on navigational bias and a focus on what is known, and Stellefson et al [39] found that many health professional college students are rather unconfident when evaluating information from the Internet. Furthermore, a younger age and more Internet experience might enhance operational skills, but previous studies have found that strategic eHealth literacy problems are still frequently present among students who grew up using the Internet [16,17,19]. Some exploratory analysis on our data, however, indicated that patients with a higher educational level, younger age, and higher perceived Internet skills completed more assignments, performed better, and encountered fewer problems. All in all, it should be acknowledged that a broad range of eHealth literacy problems exist, but future research should focus on which groups of people struggle with specific categories of eHealth literacy problems.

From our results, it seemed that several shifts were necessary to make online information, communication, and participation services more beneficial in rheumatology. First of all, the problems that were observed in these studies cannot solely be attributed to the patients’ skills, since the usability of Health 1.0 and Health 2.0 applications also plays a major role in overcoming operation, navigation, and information problems. Websites and interactive applications should be designed in a user-centered manner to overcome problems that many novice Internet users may experience [2,10,23,40]. In order to reach this, guidelines should be followed to focus on keeping a website plain and simple regarding navigation structures and usage of buttons [41]. Furthermore, it is essential that texts are written on a level that is understandable for the majority of the population [42]. Usability of Health 2.0 applications could, moreover, be increased by explaining their function, use, and privacy procedures in the application itself, for example using demonstration videos. Finally, to ensure that usability goals are reached, websites should be tested with representative end-users in several stages of the development [43,44]. Health care organizations could also play a role in tuning the level of online applications to patients’ eHealth literacy, by developing websites and Web portals which provide reliable and valuable information [25]. Second, patients should be informed and educated about proper use and protection of privacy on the Web. This could be realized in (online) eHealth literacy courses, which seem to be promising [45,46]. Third, tools could be developed which care providers could use in consult, in order to gain attention among patients for both the possibilities and the risks of the Internet [47]. A final necessity that follows from our results, is an eHealth literacy measurement instrument that can identify a broad range of skills. The eHEALS scale by Norman [48] or the Functional, Communicative, and Critical health literacy scale by Ishikawa [49] offer good starting points for this area, provided that Health 2.0 skills measures are added.

In conclusion, patients with rheumatic diseases often seek online disease-related information, and online interactive applications that help patients to get more involved in learning and caring for their disease are promising. However, the majority of the patients lack the skills to use both Health 1.0 and Health 2.0 properly for their own benefit. Problems include operating, navigating, searching the Internet, critically evaluating online content, and adding personal content while keeping privacy in mind. To decrease these problems, changes should be made in the design process of websites and online applications. Awareness, measurement, and education in eHealth literacy should also be increased.

## Multimedia Appendix 1

Completed tasks, performance, and number of encountered problems related to education level, age, and perceived Internet skills in studies 1 and 2.
