# Supplementary material for: Measuring Actual eHealth Literacy Among Patients With Rheumatic Diseases: a Qualitative Analysis of Problems Encountered Using Health 1.0 and Health 2.0 Applications
Source: J Med Internet Res. 2013 Feb 11;15(2):e27. doi: 10.2196/jmir.2428 (PMC3636295; doi:10.2196/jmir.2428)
Supplement: Supplementary file 1 [file jmir_v15i2e27_app1.pdf]

Table 7a: Completed tasks, performance and number of encountered problems related to education level, age and perceived Internet skills in study 1 (6 tasks) (n = 15)

Participant characteristics	N independently completed tasks (median)	N performed tasks (median)			N encountered problems
		Poor	Reasonable	Good	
Education level					
Low (n=6)	3.5	1	2	2	15
Middle to high (n=9)	4	0	2	2	19
Age					
Young (<50, n=4)	5	0	1.5	2.5	12
Elder (≥50, n=11)	3	1	2	2	19
Perceived Internet skills					
Poor to average (n=6)	3	0	3	2	19
Good to excellent (n=9)	4	1	1	4.5	10

Table 7b: Completed tasks, performance and number of encountered problems related to education level, age and perceived Internet skills in study 2 (10 tasks) (n = 16)

Participant characteristics	N independently completed tasks (median)	N performed tasks (median)			N encountered problems
		Poor	Reasonable	Good	
Education level					
Low (n=6)	4.5	4	3.5	0.5	20
Middle to high (n=10)	10	0	2.5	6.5	8.5
Age					
Young (<50, n=9)	10	0	2	6	8
Elder (≥50, n=7)	6	3	3	1	15
Perceived Internet skills					
Poor to average (n=9)	6	1	3	4	14
Good to excellent (n=7)	10	0	2	6	9
